# *Acori Tatarinowii* Rhizoma: A comprehensive review of its chemical composition, pharmacology, pharmacokinetics and toxicity

**DOI:** 10.3389/fphar.2023.1090526

**Published:** 2023-03-16

**Authors:** Jianxia Wen, Yi Yang, Junjie Hao

**Affiliations:** ^1^ School of Food and Bioengineering, Food Microbiology Key Laboratory of Sichuan Province, Xihua University, Chengdu, China; ^2^ College of Pharmaceutical Science, Yunnan University of Chinese Medicine, Kunming, China

**Keywords:** Acori tatarinowii rhizoma, chemical composition, pharmacology, pharmacokinetics, toxicity, review

## Abstract

*Acori Tatarinowii* Rhizoma (ATR, *Shi Chang Pu* in Chinese), a natural product with multiple targets in various diseases. This review provides the comprehensive summary of the chemical composition, pharmacological effects, pharmacokinetics parameters and toxicity of ATR. The results indicated that ATR possesses a wide spectrum of chemical composition, including volatile oil, terpenoids, organic acids, flavonoids, amino acids, lignin, carbohydrates and so on. Accumulating evidence from various studies has shown that ATR exerts a wide range of pharmacological properties, including protecting nerve cells, alleviating learning and memory impairment, anti-ischemic, anti-myocardial ischemia, anti-arrhythmic, anti-tumor, anti-bacterial, and anti-oxidant activities. Currently, ATR is widely used in the central nervous system, cardiovascular system, gastrointestinal digestive system, respiratory system in China, and for the treatment of epilepsy, depression, amnesia, consciousness, anxiety, insomnia, aphasia, tinnitus, cancers, dementia, stroke, skin diseases, and other complex diseases. Pharmacokinetic studies indicated that β-asarone, α-asarone, *cis*-methylisoeugenol, and asarylaldehyde, the active components of ATR, were absorbed slowly after oral administration of ATR. Moreover, toxicity studies have suggested that ATR has no carcinogenic, teratogenic and mutagenic toxicity. Nevertheless, long term or high-dose toxicity testing in animals to explore the acute and chronic toxicity of *acori Tatarinowii* Rhizoma is still lacking. In view of good pharmacological activities, ATR is expected to be a potential drug candidate for the treatment of Alzheimer’s disease, depression, or ulcerative colitis. However, further studies are needed to elucidate its chemical composition, pharmacological effects, molecular mechanisms and targets, improve its oral bioavailability, and clarify its potential toxicity.

## Introduction

*Acori Tatarinowii* Rhizoma (ATR, *Shi Chang Pu* in Chinese) is the dried rhizome of *Acorus tatarinowii* Schott., a perennial herb of the Araceae Juss (Yan et al., 2020b). It is first recorded in the classic works of traditional Chinese medicine “Shen Nong’s Materia Medica,” and is listed as a top grade. The effects of ATR are mainly to resuscitate, calm the mind, resolve *shi* (dampness) and harmonize the *wei* (stomach) (Lam et al., 2016b). Clinically, ATR is widely used for neurological disorders, cardiovascular system, gastrointestinal digestive system, respiratory system in China (Lam et al., 2016b; Li et al., 2018a), and for the treatment of epilepsy, depression, amnesia, consciousness, anxiety, insomnia, aphasia, tinnitus, cancers, dementia, stroke, skin diseases, and other complex diseases (Lee et al., 2004; Liu et al., 2013; Lam et al., 2019; Li J. et al., 2021). In recent years, its pharmacological research has shown that ATR has a variety of pharmacological effects, including anti-epileptic, sedative, hypnotic, anti-convulsant, anti-tussive, anti-asthmatic, anti-oxidant, anti-tumor and so on (Wu et al., 2015; Lam et al., 2017a; Fu et al., 2020; Shi et al., 2020; Zhang W. et al., 2022). Previous studies indicated that ATR is promising as a potential drug candidate for the treatment of Alzheimer’s disease (AD), depression, or ulcerative colitis. In view of the exact clinical efficacy of ATR and the continuous discovery of new pharmacological activities and active ingredients, it has been widely concerned worldwide in recent years and has become one of the hot researched Chinese medicine varieties in the medical field.

The chemical composition and pharmacological effects of ATR have been extensively reported over the past few decades, and its pharmacokinetics and toxicity have also been studied in varying degrees. However, most of the previous reports are scattered, lacking systematic summary and induction of ATR. Therefore, this review aims to provide a comprehensive summary and discussion of its chemical composition, pharmacology, pharmacokinetics and toxicity characteristics, thereby contributing to the further clinical practice and application of ATR.

## Chemical composition of ATR

The chemical composition of ATR are mainly volatile components and non-volatile components. The ATR essential oil (ATEO) is considered to be the active component of ATR, and the content of ATEO is the only indicator for the determination of ATR content. At present, there are various researches on volatile parts and relatively less research on non-volatile parts. The volatile components are relatively complex, and the main structural types are phenylpropanoids (simple phenylpropanoids, lignans and coumarins) and terpenoids (monoterpenes, sesquiterpenes, diterpenoids and triterpenes). Non-volatile components are mainly alkaloids, aldehydes and acids, quinones and ketones, sterols, amino acids, and carbohydrates. The results of the ATR chemical composition study will contribute to the development of its quality research.

### Volatile composition

**FIGURE 1:**
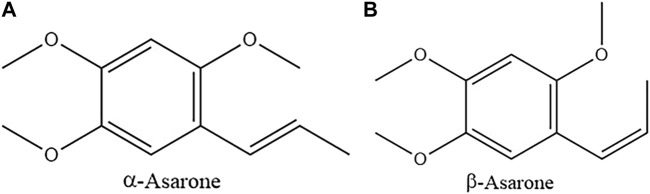
The chemical structure of α-asarone (PubChem CID: 636822) **(A)**, and β-asarone (PubChem CID: 5281758) **(B)**.

Researchers used analytical testing techniques such as chromatography and GC-MS to analyze the chemical components of ATR from different origins, different batches, different extraction methods and different parts. Previous studies indicated that the main chemical constituents in ATR were volatile oils, which are the important indicator for quality evaluation of ATR. α-Asarone and β-asarone accounted for 95% of ATR volatile oils and were identified as characteristic components (Figure 1) (Lam et al., 2016a). The “Pharmacopoeia of The People’s Republic of China” (2020 Edition) records that the volatile oil content of ATR should not be less than 1.0% (mL/g). Currently, multiple kinds of volatile oil components were found in ATR (Table 1).

**TABLE 1:** The volatile oil composition in ATR.

Compounds	Formula	Molecular weight	CAS No.	References
o-Cymene	C_10_H_14_	134.218	527-84-4	Lam et al. (2017a), Yan et al. (2020a)
α-Pinene	C_10_H_16_	136.234	2,437-95-8	Xiaojuan Zhang et al. (2015), Lam et al. (2017a), Liu et al. (2017), Yan et al. (2020a)
β-Pinene	C_10_H_16_	136.234	127-91-3	Xiaojuan Zhang et al. (201b), Yan et al. (2020a)
(−)-Camphene	C_10_H_16_	136.234	5,794-04-7	Xiaojuan Zhang et al. (2015), Yan et al. (2020a)
3-Carene	C_10_H_16_	136.234	13,466-78-9	Wu et al. (2013)
Limonene	C_10_H_16_	136.234	138-86-3	Lam et al. (2017a), Yan et al. (2020a)
α-Terpinene	C_10_H_16_	136.234	99-86-5	Lam et al. (2017a), Yan et al. (2020a)
γ-Terpinene	C_10_H_16_	136.234	99-85-4	Xiaojuan Zhang et al. (2015), Yan et al. (2020a)
Estragole	C_10_H_12_O	148.202	140-67-0	Lam et al. (2017a), Yan et al. (2020a)
Camphor	C_10_H_16_O	152.233	76-22-2	Xiaojuan Zhang et al. (2015), Lam et al. (2017a)
(−)-Borneol	C_10_H_18_O	154.249	464-45-9	Xiaojuan Zhang et al. (2015), Zhang et al. (2020)
α-terpineol	C_10_H_18_O	154.249	98-55-5	Yan et al. (2020a), Zhang et al. (2020)
Cineole	C_10_H_18_O	154.249	406-67-7	Wu et al. (2013)
4-Terpineol	C_10_H_18_O	154.249	562-74-3	Liu et al. (2006), Wu et al. (2013), Shi et al. (2021)
Linalool	C_10_H_18_O	154.249	78-70-6	Xiaojuan Zhang et al. (2015), Lam et al. (2017a), Yan et al. (2020a)
Eucalyptol	C_10_H_18_O	154.249	470-82-6	Xiaojuan Zhang et al. (2015), Yan et al. (2020a)
Menthol	C_10_H_20_O	156.265	1,490-04-6	Wu et al. (2013), Shi et al. (2021)
Methyl eugenol	C_11_H_14_O_2_	178.228	93-15-2	Liu et al. (2006), Wu et al. (2013), Tang et al. (2014), Wang et al. (2015), Shi et al. (2021)
*cis*-methylisoeugenol	C_11_H_14_O_2_	178.228	6,380-24-1	Xiaojuan Zhang et al. (2015), Lam et al. (2017a), Liu et al. (2017), Song et al. (2018), Yan et al. (2020a), Zhang et al. (2020)
trans-methylisoeugenol	C_11_H_14_O_2_	178.228	6,379-72-2	Lam et al. (2017a), Liu et al. (2017), Xiaojuan Zhang et al. (2015), Zhang et al. (2020)
2,4,5-Trimethoxybenzaldehyde	C_10_H_12_O_4_	196.200	4,460-86-0	Wu et al. (2017), Shi et al. (2021)
α-Calacorene	C_15_H_20_	200.319	21,391-99-1	Lam et al. (2017a), Liu et al. (2017), Wu et al. (2017), Yan et al. (2020a), Shi et al. (2021)
(−)-β-Caryophyllene	C_15_H_24_	204.351	87-44-5	Xiaojuan Zhang et al. (2015), Lam et al. (2017a), Yan et al. (2020a)
(+)-β-Caryophyllene	C_15_H_24_	204.351	87-44-5	Xiaojuan Zhang et al. (2015), Lam et al. (2017a), Zhang et al. (2020)
α-Caryophyllene	C_15_H_24_	204.351	6,753-98-6	Yan et al. (2020a)
β-Caryophyllene	C_15_H_24_	204.351	87-44-5	Liu et al. (2006), Wu et al. (2013), Tang et al. (2014), Shi et al. (2021)
α-Gurjunene	C_15_H_24_	204.351	489-40-7	Wu et al. (2013), Tang et al. (2014), Shi et al. (2021)
γ-Gurjunene	C_15_H_24_	204.351	22,567-17-5	Wu et al. (2013), Wang et al. (2015), Shi et al. (2021)
α-Cadinene	C_15_H_24_	204.351	24,406-05-1	Wu et al. (2013), Shi et al. (2021)
β-Cadinene	C_15_H_24_	204.351	523-47-7	Wu et al. (2013), Lam et al. (2017a), Shi et al. (2021)
γ-Cadinene	C_15_H_24_	204.351	39,029-41-9	Wu et al. (2013), Lam et al. (2017a), Shi et al. (2021)
δ-Cadinene	C_15_H_24_	204.351	483-76-1	Wu et al. (2013), Tang et al. (2014), Xiaojuan Zhang et al. (2015), Wang et al. (2015), Liu et al. (2017), Yan et al. (2020a), Shi et al. (2021)
α-Patchoulene	C_15_H_24_	204.351	560-32-7	Lam et al. (2017a), Yan et al. (2020a)
α-Panasinsen	C_15_H_24_	204.351	56,633-28-4	Yan et al. (2020a)
α-Longipinene	C_15_H_24_	204.351	5,989-08-2	Lam et al. (2017a), Yan et al. (2020a)
α-Acoradiene	C_15_H_24_	204.351	28,400-13-7	Yan et al. (2020a)
Longifolene	C_15_H_24_	204.351	475-20-7	Yan et al. (2020a)
Longicyclene	C_15_H_24_	204.351	1,137-12-8	Xiaojuan Zhang et al. (2015), Lam et al. (2017a), Song et al. (2018), Yan et al. (2020a)
β-Elemene	C_15_H_24_	204.351	515-13-9	Xiaojuan Zhang et al. (2015), Lam et al. (2017a), Yan et al. (2020a)
δ-Elemene	C_15_H_24_	204.351	20,307-84-0	Xiaojuan Zhang et al. (2015), Yan et al. (2020a)
Calarene	C_15_H_24_	204.351	17,334-55-3	Xiaojuan Zhang et al. (2015), Song et al. (2018), Yan et al. (2020a)
Germacrene D	C_15_H_24_	204.351	317,819-80-0	Xiaojuan Zhang et al. (2015), Yan et al. (2020a)
α-Asarone	C_12_H_16_O_3_	208.254	2,883-98-9	Zhu et al. (2010), Xiaojuan Zhang et al. (2015), Lam et al. (2017a), Liu et al. (2017), Lam et al. (2019), Yan et al. (2020a), Zhang et al. (2020)
β-Asarone	C_12_H_16_O_3_	208.254	5,273-86-9	Zhu et al. (2010), Xiaojuan Zhang et al. (2015), Lam et al. (2017a), Liu et al. (2017), Li et al. (2018a), Song et al. (2018), Lam et al. (2019), Yan et al. (2020a), Zhang et al. (2020)
γ-Asarone	C_12_H_16_O_3_	208.254	5,353-15-1	Xiaojuan Zhang et al. (2015), Lam et al. (2017a), Liu et al. (2017), Song et al. (2018), Yan et al. (2020a)
Elemisin	C_12_H_16_O_3_	208.254	487-11-6	Tang et al. (2014), Wang et al. (2015), Shi et al. (2021)
Shyobunone	C_15_H_24_O	220.350	21,698-44-2	Xiaojuan Zhang et al. (2015), Lam et al. (2017a), Yan et al. (2020a)
Isoshyobunone	C_15_H_24_O	220.350	21,698-46-4	Lam et al. (2017a), Liu et al. (2017), Yan et al. (2020a)
Caryophyllene oxide	C_15_H_24_O	220.350	1,139-30-6	Song et al. (2018), Yan et al. (2020a)
Eremophila ketone	C_15_H_24_O	220.350	158,930-41-7	Yan et al. (2020a)
Spathulenol	C_15_H_24_O	220.350	6,750-60-3	Lam et al. (2017a), Yan et al. (2020a)
α-Cadinol	C_15_H_26_O	222.366	481-34-5	Xiaojuan Zhang et al. (2015), Lam et al. (2017a), Yan et al. (2020a)
α-Bisabolol	C_15_H_26_O	222.366	515-69-5	Liu et al. (2006), Shi et al. (2021)
Viridiflorol	C_15_H_26_O	222.366	51,371-47-2	Yan et al. (2020a)
Dihydroagarofuran	C_15_H_26_O	222.366	20,053-66-1	Wu et al. (2013), Shi et al. (2021)
Aihydroagarofuran	C_15_H_26_O	222.366	5,956-09-2	Yan et al. (2020a)
Elemol	C_15_H_26_O	222.366	639-99-6	Xiaojuan Zhang et al. (2015), Yan et al. (2020a)
Germacrene D-4-ol	C_15_H_26_O	222.366	74,841-87-5	Yan et al. (2020a)
Isocalamendiol	C_15_H_26_O_2_	238.366	25,330-21-6	Wu et al. (2013), Shi et al. (2021)
Linoleic acid	C_18_H_32_O_2_	280.445	60-33-3	Dong et al. (2007), Li et al. (2013), Wang et al. (2015), Shi et al. (2021)
Elaidic Acid	C18H34O2	282.461	112-79-8	Wang et al. (2015)

### Non-volatile components

Most of the non-volatile components of ATR are in its aqueous extraction and organic solvent extraction parts, and the *n*-butanol fraction of the ethanol extract can be separated and purified to obtain alkaloids, phenylpropanoid derivatives, and furan compounds, pyrone compounds, organic acid compounds and diterpene glycoside compounds (Dong et al., 2008; Yang et al., 2021). This study summarizes the terpenoids, organic acids, and flavonoids components in ATR. Among them, the terpenoids in ATR include shyobunone, acoronene, cycloartenol, lupeol, and daucosterol (Table 2). The organic acids in ATR include fumaric acid, benzoic acid, nicotinic acid, 4-hydroxybenzoic acid, protocatechuic acid, vanillic acid, suberic acid, caffeic acid. (Table 3). The flavonoids in ATR include astragalin, rhodionin, rhoifolin, kaempferol-3-rutinoside (Table 4).

**TABLE 2:** The terpenoids composition in ATR.

Compounds	Formula	Molecular weight	CAS No.	References
Shyobunone	C_15_H_24_O	220.350	21,698-44-2	Ni and Yu (2013), Shi et al. (2021)
Acoronene	C_15_H_22_O_2_	234.33	33,983-45-8	Ni and Yu (2013), Shi et al. (2021)
(3beta,24S)-stigmast-5-en-3-ol	C_29_H_50_O	414.707	83-47-6	Ni and Yu (2013), Shi et al. (2021)
Cycloartenol	C_30_H_50_O	426.717	469-38-5	Ni and Yu (2013), Song et al. (2018), Shi et al. (2021)
Lupeol	C_30_H_50_O	426.717	545-47-1	Ni and Yu (2013), Shi et al. (2021)
Daucosterol	C_35_H_60_O_6_	576.847	474-58-8	Ni and Yu (2013), Shi et al. (2021)

**TABLE 3:** The organic acids composition in ATR.

Compounds	Formula	Molecular weight	CAS No.	References
Fumaric acid	C_4_H_4_O_4_	116.072	110-17-8	Dong et al. (2007), Li et al. (2013), Shi et al. (2021)
Benzoic acid	C_7_H_6_O_2_	122.120	65-85-0	Dong et al. (2007), Li et al. (2013), Shi et al. (2021)
Nicotinic acid	C_6_H_5_NO_2_	123.110	59-67-6	Dong et al. (2007), Li et al. (2013), Shi et al. (2021)
4-Hydroxybenzoic acid	C_7_H_6_O_3_	138.121	99-96-7	Dong et al. (2007), Li et al. (2013), Shi et al. (2021)
Protocatechuic acid	C_7_H_6_O_4_	154.120	99-50-3	Dong et al. (2007), Li et al. (2013), Shi et al. (2021)
Vanillic acid	C_8_H_8_O_4_	168.147	121-34-6	Dong et al. (2007), Li et al. (2013), Shi et al. (2021)
Suberic acid	C_8_H_14_O_4_	174.194	505-48-6	Dong et al. (2007), Li et al. (2013), Shi et al. (2021)
Caffeic acid	C_9_H_8_O_4_	180.157	331-39-5	Dong et al. (2007), Li et al. (2013), Shi et al. (2021)
Ferulic acid	C_10_H_10_O_4_	194.184	537-98-4	Dong et al. (2007), Li et al. (2013), Shi et al. (2021)
Myristoleic acid	C_14_H_26_O_2_	226.355	544-64-9	Dong et al. (2007), Li et al. (2013), Shi et al. (2021)
Palmitic acid	C_16_H_32_O_2_	256.424	60,605-23-4	Dong et al. (2007), Li et al. (2013), Wu et al. (2013), Shi et al. (2021)
Cryptochlorogenic acid	C_16_H_18_O_9_	354.309	905-99-7	Dong et al. (2007), Li et al. (2013), Shi et al. (2021)

**TABLE 4:** The flavonoids composition in ATR.

Compounds	Formula	Molecular weight	CAS No.	References
Astragalin	C_21_H_20_O_11_	448.377	480-10-4	Tong and Cheng (2011), Shi et al. (2021)
Rhodionin	C_21_H_20_O_11_	448.377	85,571-15-9	Tong and Cheng (2011), Shi et al. (2021)
Rhoifolin	C_27_H_30_O_14_	578.519	17,306-46-6	Tong and Cheng (2011), Shi et al. (2021)
kaempferol-3-rutinoside	C_27_H_30_O_15_	594.518	17,650-84-9	Tong and Cheng (2011), Shi et al. (2021)

In addition to the above components, ATR also contains amino acids, lignin, carbohydrates and other chemical components. Among the amino acids, both human essential amino acids, and human semi-essential amino acids are contained. In addition, ATR also contains glucose and trace elements, alkaloids, etc. (Hu et al., 2019). The lignan components include bergapten, marmesin, eudesmin and so on. Its carbohydrate components are mainly glucose, maltose, fructose and mannose (Shi et al., 2021). Zhang et al., focused on large molecular components such as polysaccharides in ATR, speculating that the immune activity of ATR may be related to active polysaccharide components. In their study, DEAE-52 cellulose and Sephadex G-100 column chromatography were used to separate and purify polysaccharide from water chestnut base extracted polysaccharide. The eluent with absorbance greater than 0.3 in polysaccharide is collected and freeze-dried, and is named RATAPW. The average molecular weight of RATAPW was 2.51 × 10^4^ Da, and the total carbohydrate content of RATAPW was 98.23% ± 0.29%. Monosaccharide composition, methylation and nuclear magnetic resonance (NMR) analysis showed that the polysaccharide was α-1,4-glucan with short α-1,6 branches (Zhang Y. et al., 2022).

## Pharmacological effects of ATR

### Effects on the central nervous system

The regulation of ATR on the central nervous system is particularly significant and has a bidirectional regulatory effect. Modern research shows that ATR and its active components α-asarone and β-asarone have effects on calming the nerves, anti-epileptic, anti-depression, anti-AD, anti-convulsant, and anti-dementia (Yang et al., 2021). Moreover, ATR and its active components also have a good protective effect on brain tissue and nerve cells, and can improve the permeability of the blood-brain barrier (BBB), promote the entry of other substances into the brain tissue through the BBB, and improve the blood concentration and bioavailability of drugs in the brain tissue (Jiang et al., 2018). The high safety and obvious curative effect of ATR in the treatment of central nervous system disease make it have a good development and application prospect. However, studies also showed that α-asarone and β-asarone could shrink the endothelial cells and loosen the tight connection, thus increasing the permeability of the BBB and assisting the drug to enter the brain tissue, which is in contradiction with the above research mechanism (Huang et al., 2016). Thus, whether asarone has protective effect on BBB needs further research to confirm.

#### Protective effect on nerve cells

α-Asarone and β-asarone are considered to be the main active components of ATR as a Chinese herbal medicine, and both can be potential candidates for drug development in neurodegenerative diseases. Lam et al., found that when performed to cultured rat astrocytes, ATR volatile oil, α-asarone and β-asarone could dose-dependently stimulate the expression and secretion of neurotrophic factor, namely, nerve growth factor (NGF), brain-derived neurotrophic factor (BDNF) and glial-derived neurotrophic factor (GDNF) in cultured PC12 cells (Lam et al., 2016a; Lam et al., 2017a; Lam et al., 2019). In addition, β-asarone could protect PC12 cells from amyloid beta (Aβ)-induced damage and regulates the expression of autophagy factors. Simultaneously, the cytoprotective effects of ATR oil, α-asarone and β-asarone on tert-butyl hydroperoxide (tBHP)-induced astrocyte injury were also revealed, which reduced tBHP-induced accumulation of reactive oxygen species (ROS) in astrocytes. Moreover, the activity of the transfected anti-oxidant response element (ARE) promoter construct (pARE-Luc) and the mRNAs encoding anti-oxidant enzymes were regulated by ATR oil and asarones, whose gene expression may be mediated by Akt phosphorylation (Lam et al., 2017a; Lam et al., 2017b). Moreover, ATR volatile oil, α-asarone, or β-asarone induces the transcriptional activation of neurofilament promoters and potentiates NGF-induced neurite outgrowth and neurofilament expression. Mechanism research found ATR volatile oil, α-asarone or β-asarone, induces phosphorylation of cAMP-response element binding protein (CREB) and cAMP-mediated transcriptional activity. Above all, α-asarone and β-asarone synergistically increase transcriptional activation of neurofilament promoters (Lam et al., 2016a). α-Asarone and β-asarone, as the main active ingredient in volatile oil, play a wide and important pharmacological role protecting nerve cells. Asarones or ATR volatile oil promoting axons might help to find potential agents for treating various neurodegenerative diseases.

**FIGURE 2:**
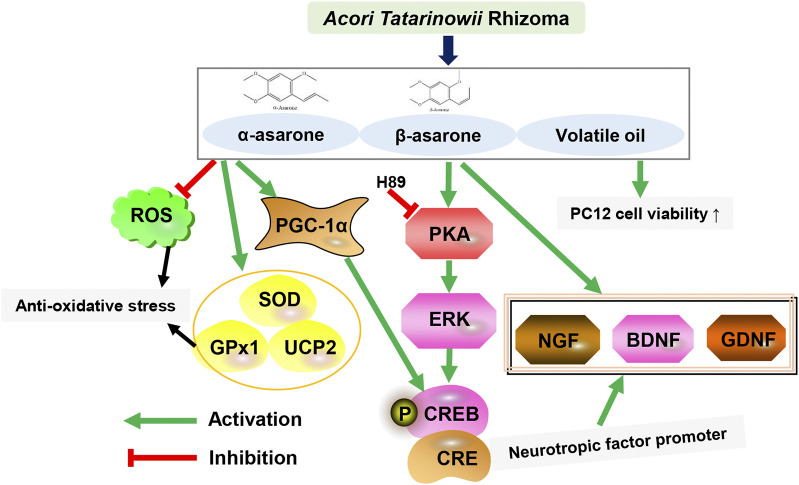
The main biological activities and potential mechanism of ATR on the central nervous system.

β-Asarone could improve the degree of cerebral edema in rats with ischemia-reperfusion injury, and it have effects on reducing oxidative stress injury, inhibiting brain cell apoptosis, regulating amino acid levels and excitatory amino acid toxicity (Huang et al., 2020). Furthermore, it could increase the expression levels of Glutamic acid, Aspartic acid, and gamma-aminobutyric acid, and improve cerebral ischemia tolerance (Ke and Fang, 2003), thereby reducing the damage to neurons caused by the above substances during cerebral ischemia-reperfusion, thus playing a role in brain protection. Li et al., established a PC12 cell line with APPswe-overexpressing as a cellular model of Aβ-induced injury and assessed autophagic flux-related proteins as well as the number and morphology of autophagosomes and autolysosomes (Li Z. et al., 2021). The results indicated that β-asarone could reduce the expression levels of Beclin-1, p62, LC3-II, and Aβ_1-42_. β-Asarone decreased the number of autophagosomes and increased the number of autolysosomes. Studies have shown that β-asarone can protect PC12 cells from Aβ-induced damage by promoting autophagic flux, which can be achieved by enhancing autophagosome-lysosome fusion and/or lysosomal function. In addition, the essential oil is also considered as the active fraction of ATR. To investigate the anti-oxidative stress effects of ATEO, a H_2_O_2_-stressed neuronal cell model was established by Yan et al.,. ATEO treatment increased the viability of cells affected by H_2_O_2_-mediated injury, inhibited the accumulation of ROS, and induced the expression of several anti-oxidant proteins (SOD, GPx, and UCPs). It was suggested that ATEO may effectively prevent H_2_O_2_-induced cell damage by activating CREB/peroxisome proliferator-activated receptor gamma coactivator 1-alpha (PGC-1α) signaling in PC12 cells and exerting an anti-oxidative stress effect (Yan et al., 2020b). In addition, the ATR polysaccharides could protect PC12 cells from H_2_O_2_-induced injury, which could substantially stimulate nitric oxide (NO) production and phagocytic activity in RAW264.7, and promoted splenocyte proliferation (Zhang W. et al., 2015). The results showed that ATR polysaccharide could serve as a novel natural source of anti-oxidant and immune enhancer (Table 5; Figure 2).

**TABLE 5:** Effects of ATR on the central nervous system.

Effects	Animals/cells	Experimental model	Dosage/concentrations	Pharmacological effects	Targets/pathways
AD (Li et al., 2021b)	PC12 cells	PC12 cell line with APPswe-overexpressing as a cellular model of Aβ-induced injury	72 μM β-asarone treatment for 24 h	Promote autophagic flux, enhance autophagosome-lysosome fusion and/or lysosomal function	Reduce the expression levels of Beclin-1, p62, LC3-II and Aβ_1-42_
AD (Mao et al., 2015)	C57BL/6 mice, APP/PS1 transgenic mice, adult hippocampal NPCs	APP/PS1 mice and their wild-type littermates	10 g/kg ATR, 10 mg/kg α-asarone, 30 mg/kg β-asarone in mice (*i.g.*) 0.1–1 mg/mL ATR, 0.3–10 μM α-asarone or β-asarone *in vitro*	Promote NPC proliferation and neurogenesis	activated ERK but not Akt
ROS-mediated damage in neuronal cells (Yang et al., 2021)	PC12 cells	H_2_O_2_-induced injury	1.5, 5, and 15 μg/mL for 48 h	Suppresse the accumulation of ROS and induces the expression of anti-oxidant proteins	Activation of CREB/PGC-1α signaling
Neurotrophin deficiency (Lam et al., 2016a)	PC12 cells	Low concentration NGF-induced neurite outgrowth and neurofilament expression	30 μg/mL for 48 h	Induces the transcriptional activation of neurofilament promoters and potentiates NGF-induced neurite outgrowth and neurofilament expression	Induces phosphorylation of CREB and cAMP-mediated transcriptional activity
Anti-oxidative and immunopotentiating (Zhang et al., 2015a)	Mice, PC12 cells	H_2_O_2_-induced PC12 cell death	10, 100, 200 μg/mL for 12 h	Promotes splenocyte proliferation, anti-oxidant	Stimulates NO production and phagocytic activity
Defective neurotrophic factor expression (Lam et al., 2017a; Lam et al., 2019)	Astrocytes	Neurotrophic factor expression partially blocked by PKA inhibitor, H89	50 μM	Stimulates the expression and secretion of neurotrophic factors	PKA signaling
Astrocytes cell injury (Lam et al., 2017a; Lam et al., 2017b)	Astrocytes	tBHP-induced intracellular ROS accumulation	0.5–15 μg/mL α-asarone, β-asarone or ATR oil	Reduce astrocytes cell injury and ROS accumulation	Akt phosphorylation
AD (Ning et al., 2021)	Mice	Scopolamine-induced cognitive impairment (*i.p.*)	1.56, 6.24 g/kg/d PA (*i.g.*) 0.78, 3.12 g/kg/d ATR (*i.g.*)	Increase neurotransmitter concentrations in the hippocampus, increase synapse-associated proteins to alleviate cognitive deficits	Activated BDNF/ERK/CRE signaling pathway, and increased p90RSK and PSD95 protein expression

Notes: ATR, acori tatarinowii rhizoma; AD, anti-Alzheimer’s disease; ROS, reactive oxygen species; NPC, neural progenitor cell; NGF, nerve growth factor; ERK, extracellular signal-regulated kinase; CREB, cAMP-response element binding protein; BDNF, brain-derived neurotrophic factor.

#### Effects on learning and memory impairment

ATR extract and its active ingredient, asarones could promote aberrant neural progenitor cell (NPC) proliferation. Oral administration of ATR enhanced NPC proliferation and neurogenesis in the hippocampus of adult and aged mice as well as in transgenic AD model mice. ATR and its components also enhanced the proliferation of cultured NPCs *in vitro*. Mechanistic studies have shown that ATR and asarones could activate the key kinase cascades in neurogenesis, extracellular signal-regulated kinase (ERK), but not Akt. Studies have shown that oral administration of ATR and asarones could be used as preventive and regenerative therapeutics to promote neurogenesis against age-related neurodegeneration and neurodegenerative disorders (Mao et al., 2015). Ning et al. (2021) explored the protective effect of Polygoni Multiflori Radix Praeparata (PMRP) combined with ATR (PA) on scopolamine-induced cognitive impairment in mice and its potential mechanism. PA was found to increase the concentration of neurotransmitters in the hippocampus, activate the BDNF/ERK/CREB signaling pathway, and increase the p90 ribosome expression of S6 kinase (p90RSK) and postsynaptic density (PSD) 95 proteins. Therefore, PA alleviates cognitive deficits by enhancing synapse-associated proteins, suggesting its therapeutic potential for the treatment of aging-related diseases such as AD (Table 5; Figure 2).

System biology research could predict the active components of ATR, as well as its corresponding targets and potential mechanism, and provide new ideas and directions for further research on the mechanism of ATR. Studies have (Song et al., 2018; Liu et al., 2020; Zhang et al., 2020) revealed a multicomponent synergistic mechanism and molecular targets of ATR in AD using a system pharmacology strategy. The results showed that the active components of ATR were involved in cancer, inflammation, cell metabolism, metabolic pathways and other related biological processes (Zhang et al., 2020), and were associated with a variety of predicted targets, such as amyloid precursor protein (APP), estrogen receptor 1 (ESR1), peroxisome proliferator activated receptor gamma (PPARG), androgen receptor (AR), muscarinic acetylcholine receptor M1 (CHRM1), caspase 3 (CASP3), janus kinase 2 (JAK2), mitogen-activated protein kinase 14 (MAPK14), prostaglandin G/H synthase 1 (PTGS1), protein kinase cAMP-activated catalytic subunit alpha (PRKACA). Moreover, most compounds in ATR have anti-fibrillar amyloid plaque, anti-inflammatory and anti-tau phosphorylation effects (Song et al., 2018). The potential mechanisms were found to be mainly involved in phosphoinositide 3-kinase (PI3K)-Akt, JAK2, MAPK, protein tyrosine phosphatase non-receptor type 1 (PTPN1) signaling pathway, neuroactive ligand-receptor interaction, as well as fluid shear stress and atherosclerosis (Liu et al., 2020; Zhang et al., 2020). ATR mainly acts on the kinase domain receptor (*KDR*) gene. Organ distribution showed that the targets of active ingredients were mainly located in AD-related whole blood, heart, liver, brain and muscle (Song et al., 2018; Liu et al., 2020). The network pharmacology technology has been systematically applied to the study of the target and potential mechanism corresponding to the active components of ATR, providing a new idea and direction for further study of the mechanism of ATR in AD. These findings suggested that ESR1, JAK2, PRKACA, and PTPN1 were considered as therapeutic targets for further research on ATR treatment of AD. However, more molecular biological methods are needed to demonstrate these targets.

### Effects on the cardiovascular system

In recent years, the in-depth development and application of ATR in the cardiovascular field has become a research hotspot. ATR is widely used in the prevention and treatment of cardiovascular diseases such as coronary heart disease, hypertension and hyperlipidemia due to its multi-channel, multi-target and comprehensive regulation characteristics in the treatment of diseases. A series of *in vitro* studies have shown that ATR and its chemical components can effectively protect vascular endothelium and cardiomyocytes, and play an important role in anti-platelet aggregation, improving blood rheology, regulating blood lipids, resisting arrhythmia, lowering blood pressure, resisting myocardial hypertrophy and atherosclerosis. The current research is mainly manifested in the following aspects.

#### Anti-myocardial ischemia effect

Myocardial ischemia-reperfusion injury refers to the interruption of myocardial blood supply in a short period of time, and the restoration of blood supply within a certain period of time. This is an unavoidable anomaly that is clearly contrary to the purpose of treatment (Tsao et al., 2022). The volatile oil of ATR and β-asarone have anti-myocardial ischemia effects, which can reduce the levels of endothelin (ET), calcitonin gene-related peptide (CGRP), and norepinephrine (NE) in rats with myocardial ischemia, and increase NO level. In addition, it could increase serum superoxide dismutase (SOD) levels, decreased malondialdehyde (MDA) and creatine kinase (CK) levels (Zhong et al., 2019). Furthermore, Wang et al., used sodium dithionite (Na_2_S_2_O_4_) to induce myocardial ischemia/reperfusion injury (MI/RI) in cardiomyocytes, and explored the protective effect of the active ingredient β-asarone of ATR on myocardial cells from ischemia-reperfusion injury. It was found that β-asarone could significantly improve cell viability, reduce the content of lactate dehydrogenase (LDH) and CK in culture medium, stabilize mitochondrial membrane potential (MMP), and effectively inhibit mitochondrial damage in cardiomyocytes. The results showed that β-asarone had a significant protective effect on MI/RI cardiomyocytes (Wang et al., 2008a; Wang et al., 2008b).

#### Anti-arrhythmic effect

ATR has a certain anti-arrhythmic effect. Studies have found that aconitine could cause atrial, ventricular premature beats or the formation atrial tachycardia, short paroxysmal ventricular tachycardia, and ventricular tachycardia in rats. Epinephrine could cause single or multi-source premature ventricular contractions, paroxysmal ventricular tachycardia in rabbits. Barium chloride can induce bidirectional ventricular tachycardia-based arrhythmia in rabbits. The production of these arrhythmias is related to changes in autonomic nerves, mediators and myocardial excitability and automaticity. Shen et al., found that the volatile oil components of ATR may antagonize aconitine-induced arrhythmia in rats. Simultaneously, it is resistant to arrhythmias induced by epinephrine or barium chloride in rabbits (Shen et al., 1993). A certain concentration of ATR volatile oil could reduce the beating frequency of myocardial cells and improve the vitality of myocardial cells, so as to achieve the purpose of anti-arrhythmia. Specifically, 100–160 mg/L volatile oil from ATR can improve the survival rate of normal cardiomyocytes. The concentration of volatile oil from ATR is 140 mg/L, the survival rate of cardiac myocytes is at the top of the parabola. When the concentration is lower than 160 mg/L, there is no abnormal change in the morphology of cardiomyocytes. However, there are phenomena such as cell synapse thinning, retraction, and cytoplasmic shrinkage while the concentration is higher than 180 mg/L (Wu et al., 2009). Therefore, the volatile oil of ATR could reduce the beating frequency and enhance the viability of cardiomyocytes in a certain concentration, but it might have a certain adverse effect on normal cardiac myocytes when the concentration is too high.

### Anti-tumor effects

The main component volatile oil of ATR, β-asarone inhibited the growth of colon cancer cells and the proliferation of gastric cancer cells (Zou et al., 2012; Wu et al., 2015). Studies have shown that 30–60 μM asarone inhibited the cell viability, proliferation, colony-forming ability, migration, invasion, and adhesion of human glioma U251 cells. It regulated the levels of key proteins involved in the death receptor pathway and mitochondrial apoptosis pathway. Simultaneously, β-asarone regulated cell cycle-related proteins and inhibited tumor growth and induces apoptosis. It inhibits epithelial-mesenchymal transition (EMT) by up-regulating E-cadherin and down-regulating vimentin, and reduces the expression of the oncogenic protein hnRNPA2/B1 in a concentration- and time-dependent manner, which may be the effect of β-asarone on glioma cell invasion and EMT (Li et al., 2018a; Li et al., 2018b). In addition, β-asarone had a significant dose-dependent inhibitory effect on the cell proliferation and induction on cell apoptosis of human gastric cancer cell lines (SGC-7901, BGC-823, and MKN-28), as well as inhibiting effect on the invasion, migration and adhesion of BGC-823 cells. Furthermore, β-asarone inhibited the gastric cancer cell growth by upregulating the expression of caspase-3, caspase-8, caspase-9, Bcl2-associated X (Bax), Bcl-2 homologous antagonist/killer (Bak), reversion-inducing cysteine-rich protein with kazal motifs (RECK), E-cadherin and downregulating MMP-2, MMP-9, MMP-14, Bcl-2, Bcl-xL, and N-cadherin (Wu et al., 2015). Tao et al. (2020) investigated the exact mechanism of β-asarone in gastric cancer. The current study showed that β-asarone had a dose-dependent inhibitory effect on three gastric cancer cell lines (MGC803, SGC7901, and MKN74) at different differentiation stages. Meanwhile, under both normoxia and CoCl2-induced hypoxia, β-asarone could induce apoptosis of gastric cancer cells and block gastric cancer cells in G2/M phase of cell cycle. Besides, it decreased LDH activity in gastric cancer cells. Mechanistically, β-asarone reduces the expression of pyruvate dehydrogenase kinase (PDK) 1, phospho(p)-PDK1, PDK4, hypoxia-inducible factor 1-α (HIF-1α), c-myc, STAT5, and p-STAT5 to influence tumor glycolysis. Ultimately, β-asarone increased chemosensitization and inhibited tumor glycolysis (Table 6). In general, there are few studies on the pharmacology and clinical application of ATR in anti-tumor, and more studies are needed to investigate the effective components, pharmacological effects and molecular biological mechanisms of ATR in anti-tumor.

**TABLE 6:** Anti-tumor effects of ATR.

Chemical composition	Animals/cells	Experimental model	Dosage/concentrations	Pharmacological effects	Biological mechanism
β-Asarone (Tao et al., 2020)	Human gastric cancer cell lines MGC803, SGC7901, and MKN74	Cocl_2_ (200 μM, 24 h) to induce hypoxia conditions while H_2_O_2_ (100 μM, 1 h) to induce peroxide condition	60 μg/mL β-asarone for 24 h	Induces apoptosis in gastric cancer cells and affects tumor glycolysis	Reduce the expression of PDK1, phospho(p)-PDK1, PDK4, HIF1α, c-myc, STAT5, and p-STAT5
β-Asarone (Li et al., 2018a)	human glioma U251 cells	-	30, 60 μM β-asarone for 48 h	Inhibits migration, invasion and adhesion of U251 cells	Regulate hnRNP A2/B1 signaling pathway
β-Asarone (Li et al., 2018b)	human glioma U251 cells	-	60–480 μM β-asarone for 48 h	Inhibits cell viability, proliferation and colony-forming ability of U251 cells	Inhibit hnRNPA2/B1-mediated signaling pathway
β-Asarone (Wu et al., 2015)	Human gastric cancer cell lines SGC-7901, BGC-823 and MKN-28	-	0.12, 0.24 mM β-asarone for 24 h	Inhibits cell proliferation and induces apoptosis, reducing its ability to invade, migrate and adhere	Upregulating caspase-3, caspase-8, caspase-9, Bax, Bak, RECK, E-cadherin and downregulating MMP-2, MMP-9, MMP-14, Bcl-2, Bcl-xL and N-cadherin

### Effects on the digestive system

The free-volatile oil decoction of ATR, total volatile oil, β-asarone and α-asarone could inhibit the spontaneous contraction of isolated rabbit intestine, antagonize the intestinal spasm caused by acetylcholine (Ach), histamine phosphate (Hist) and BaCl_2_, and enhance intestinal peristalsis in rats and intestinal propulsion in mice. In addition, these components could also promote bile secretion in rats and promote the advancement of intestinal contents in mice. The above-mentioned effect is the strongest with total volatile oil, followed by α-asarone, β-asarone, and the free-volatile oil decoction of ATR was the weakest (Hu et al., 1999). The α-asarone and β-asarone contained in the volatile oil of ATR could improve the intestinal absorption of 3,4,5-trimethoxycinnamic acid (TMCA), the active ingredient of Polygalae Radix (PR), by inhibiting the function of p-glycoprotein (P-gp) in the intestinal segment (Meng et al., 2019). At the same time, the volatile oil of calamus could inhibit the intestinal absorption of saikosaponin a and promote the intestinal absorption of ginsenosides. The mechanism of promoting absorption may be related to the inhibition of P-gp (Yang et al., 2018; Wang et al., 2019). ATR could promote digestion and regulate gastrointestinal movement. It is mainly used for stomachache and abdominal pain in clinic.

### Effects on the respiratory system

Modern research shows that ATR indeed have a therapeutic effect on respiratory diseases. Li et al., investigated the anti-asthmatic effect of β-asarone on guinea pig asthma induced by ovalbumin aerosol inhalation sensitization (Li et al., 2006). The results showed that β-asarone could prolong the asthma attack latency and fall latency of model guinea pigs by spraying and gavage. However, the effect of β-asarone in spray administration was better than that in gavage administration. Xu studied the cough-relieving, phlegm-relieving and asthmatic effects of β-asarone, the active ingredient of calamus volatile oil, through experiments such as phlegm-relieving experiments in mice, cough-relieving experiments in mice, and effects on immune organs in mice (Xu, 2007). The results showed that β-asarone can increase the excretion of phenol red, which could reduce the incubation period and the number of cough attacks in cough-induced mice. Moreover, it could increase the immune organ indices of mice.

### Anti-bacterial and anti-oxidant effects

To date, several publications have reported the anti-oxidant capacity of α-asarone, β-asarone (Mukherjee et al., 2008), and isoshyobunone, calacorene, and isocalamendiol are essential oils with anti-oxidant activity (Lubsandorzhieva et al., 2013). γ-asarone shows fungitoxicity against *Aspergillus flavus* (Varma et al., 2002). δ-Cadinene in Psidium cattleianum Sabine has anti-microbial and anti-oxidant activities (Scur et al., 2016). The volatile oil of ATR has a good inhibitory effect on *staphylococcus* epidermidis, group A *streptococcus* and *shigella* flexneri (Zheng et al., 2015). Qiu et al., found that the microwave water extract of ATR has effective anti-bacterial components (Liu and Qiu, 2012). The microwave water extract of ATR has obvious anti-bacterial effect on *staphylococcus aureus* and *pseudomonas aeruginosa*. Secondly, it has a certain inhibitory effect on *salmonella* paratyphi B, *shigella* sonnei, *staphylococcus* epidermidis, *salmonella typhi*, *acinetobacter*, *shigella* flexneri and *escherichia coli*. In addition, α-asarone could regulate the activity of matrix metalloproteinases and anti-oxidant activities (Park and Kim, 2018).

### Other pharmacological effects

The RATAPW isolated from ATR by Zhang et al., can promote the production of tumor necrosis factor alpha (TNF-α) in RAW264.7 macrophages through the nuclear factor kappa B (NF-kB) molecular signaling pathway (Zhang Y. et al., 2022). Treatment with 200 μg/mL RATAPW increased the proliferation rate of spleen lymphocytes by 38.77%. RATAPW also enhanced ConA-induced T cell and lipopolysaccharide (LPS)-induced B cell proliferation in a dose-dependent manner. The research lays the foundation for the discovery of natural polysaccharide immunomodulators or functional foods from ATR.

## Clinical application of compound prescription containing ATR

The composition of traditional Chinese medicine is relatively complex. In traditional Chinese medicine, which advocates syndrome differentiation and treatment, reasonable compatibility will make it play the advantage of multi-target simultaneous action. The compound formula with ATR as the main component will produce the effect of “1 plus one is greater than two” to a certain extent. Some compounds preparations, including Kaixin San (KXS), Xian-He-Cao-Chang-Yan formula (XHCYF), Longshengzhi capsule (LSZ), Smart Soup etc. contain ATR, which are also widely used in clinical treatment for various diseases.

### Kaixin San (KXS)

KXS is a traditional Chinese herbal preparation with memory-enhancing properties that has been used for thousands of years in the medical care of depression, senile dementia, forgetfulness, and dizziness (Zhu et al., 2013). It consists of two functional pairs of herbs: Ginseng Radix (GR), PR, ATR, and Poria cum Radix Pini (PRP) (Zhu et al., 2016a; Zhu et al., 2016b; Yan et al., 2017; Cao et al., 2018; Dong et al., 2020; Qu et al., 2021). Qu et al., found that KXS exerts anti-depressant effects in chronic unpredictable mild stress-induced depression-like mice (Qu et al., 2021). It inhibits the activation of microglia and reduces the expression of pro-inflammatory cytokines in the mouse hippocampus. Kaixin powder extract decreased lipopolysaccharide-induced expression of inflammatory factors in BV2 cells by inhibiting toll-like receptor 4/inhibitor of kappa B kinase/nuclear factor kappa-B (TLR4/IKK/NF-κB) pathway in mice BV2 microglia cell lines. (Dong et al. (2021) also investigated the anti-depressant mechanism of KXS in a rat model of chronic mild stress induced by different stress methods. The results identified 33 differentially expressed proteins: seven upregulated and 26 downregulated. Functional analysis revealed that these differentially expressed proteins are involved in synaptic plasticity, neurodevelopment, and neurogenesis. In chronic mild stress (CMS)-induced depression in rats and in H_2_O_2_-stressed astrocytes model, KXS treatment could significantly alleviate CMS-induced depression symptoms, restore neurotransmitter quality, and increase the expressions of neurotrophic factors and their corresponding receptors, promote neurogenesis (Zhu et al., 2012). Moreover, KXS had the highest tendency to increase NGF, GDNF, and BDNF expression by activating cAMP-dependent signaling pathways as well as stimulating enzymes responsible for neurotrophic factor synthesis (Zhu et al., 2013; Zhu et al., 2016b; Cao et al., 2018). These therapeutic effects might be related to the modification of Erk1/2 and CREB phosphorylation (Yan et al., 2016). The anti-depressant-like effects of KXS might be mediated by increased neurotrophic factor expression in astrocytes and weren’t dependent on estrogen receptor or protein kinase-mediated signaling (Zhu et al., 2013). KXS could significantly enhance the expression levels of synaptotagmin and PSD95 by stimulating the cAMP-dependent pathway in chronic unpredictable mild stress (CUMS)-induced depressive rats, which is beneficial to synaptogenesis by inducing synaptic expression, possibly accounting for its anti-depressant effect *in vivo* and *in vitro* (Zhu et al., 2016a). In PC12 cultures, a single application of KXS had no effect on the neuronal differentiation, but showed robust effects in enhancing NGF-induced neurite outgrowth and neurofilament expression. Enhancement by KXS is mediated through the NGF receptor, tropomyosin receptor kinase (Trk) A (Yan et al., 2017). KXS might exert anti-depressant-like effects that induce neuronal differentiation (Zhu et al., 2016b; Yan et al., 2017; Dong et al., 2020; Qu et al., 2021), which supports the clinical use of this decoction.

### Xian-He-Cao-Chang-Yan formula

Li et ai., investigated the bioactive ingredients and therapeutic mechanisms of Xian-He-Cao-Chang-Yan formula (XHCF) (composition: Agrimoniae Herba, Coptidis Rhizoma, Aucklandiae Radix, Cicadae Periostracum, ATR, and Platycodonis Radix) on dextran sulfate sodium (DSS)-induced ulcerative colitis (UC) and LPS-stimulated RAW 264.7 cells (Li J. et al., 2021). The results indicated that XHCF could effectively improve DSS-induced acute colitis. It regulates macrophage polarization and inhibits glycolysis, downregulating HK2 expression in LPS-challenged macrophages. Furthermore, XHCF enhanced the phosphorylation of adenosine 5′-monophosphate (AMP)-activated protein kinase (AMPK) both *in vivo* and *in vitro*, suggesting that AMPK is involved in XHCF function.

### Longshengzhi capsule (LSZ)

LSZ has been approved by the China Food and Drug Administration for treatment of patients with cardiovascular/cerebrovascular disease. Yin et al., determined the effect of LSZ on AD processes using double transgenic mice expressing the amyloid-β precursor protein and mutant human presenilin 1 (APP/PS1) to mimic AD (Yin et al., 2020). Studies have demonstrated anti-oxidative stress, anti-inflammatory and neuroprotective effects of LSZ in AD-like pathology, and its potential mechanism was to enhance neuronal survival in HT-22 cells by partially modulating the FAS/Bcl-2/p53 pathway.

### Smart Soup

Smart Soup is a traditional Chinese medicine formula composed of ATR, PRP, and PR, which is a typical anti-memory disorder prescription. Hou et al. (2014), evaluated the efficacy of SS on AD. Oral administration of SS ameliorates cognitive impairment in AD transgenic mice, reduces Aβ levels, delays Aβ amyloidosis, and reduces Aβ-induced brain gliosis and neuronal loss in AD mice. Consistently, SS treatment reduced amyloid-related motor dysfunction and premature death in AD transgenic flies. Mechanistic studies show that ATR exerts neuroprotective effects on anti-bodies and plays a role in the treatment of AD (Table 7).

**TABLE 7:** Clinical application of compound prescription containing ATR.

Compound prescription	Formulation	Effects	Animals/cells	Experimental model	Dosage/concentrations	Pharmacological effects	Targets/pathways
Kaixin San (Qu et al., 2021)	GR, PR, ATR, PRP	Major depressive disorder (MDD)	Mice Mice BV2 microglia cell lines	Chronic unpredictable mild stress-induced depression-like mice	3, 10 g/kg/d (*i.g.*)	Inhibits the activation of microglia and reduces the expression of pro-inflammatory cytokines	Inhibite TLR4/IKK/NF-κB pathways
Kaixin San (Dong et al., 2020)	GR, PR, ATR, PRP	Depression	Rats	different stress methods-induced chronic mild stress	600 mg/kg/d (*i.g.*)	Regulation of synaptic plasticity, neurodevelopment and neurogenesis	Synaptic plasticity, neurodevelopment, and neurogenesis-related proteins
Kaixin San (Zhu et al., 2016a)	GR, PR, ATR, PRP	Depression	Rats	CUMS-induced depressive rats	1.5, 5 g/kg/d (*i.g.*)	Beneficial for synaptogenesis	Stimulation of cAMP-dependent pathways
Kaixin San (Zhu et al., 2012)	GR, PR, ATR, PRP	Depression	Rats	CMS-induced depressive	0.9, 2.7 g/kg/d (*i.g.*)	anti-depressant-like action	Increase of neurotransmitters and expression of neurotrophic factors
Kaixin San (Cao et al., 2018)	GR, PR, ATR, PRP	AD	Mouse astrocytes	-	1–10 μg/mL for 48 h	Increases NGF and BDNF expression and stimulates enzymes responsible for neurotrophic factor synthesis	activating cAMP-dependent signaling pathway
Kaixin San (Zhu et al., 2013)	GR, PR, ATR, PRP	Depression	Cultured astrocytes	Day *in vitro* (DIV) 12	0.5–50 𝜇g/mL for 24 h	Stimulates the expression and secretion of neurotrophic factors including NGF, BDNF and GDNF	Increases neurotrophic factor expression in astrocytes
Kaixin San (Zhu et al., 2016b)	GR, PR, ATR, PRP	Depression	PC12 cells	NGF-induced neuronal differentiation in PC12 cells	0.1–10 μg/mL for 48 h	Potentiate the NGF-induced neurite outgrowth	cAMP-dependent pathway
Kaixin San (Yan et al., 2017)	GR, PR, ATR, PRP	Depression	PC12 cells	NGF-induced neuronal differentiation in PC12 cells	0–100 μg/mL for 48 h or 24 h	Robust effects in NGF-induced neurite outgrowth and neurofilament expression	Trk A signaling
Kaixin San (Yan et al., 2016)	GR, PR, ATR, PRP	Depression	Rats, cultured neurons and astrocytes	CMS-induced depressive rats and H_2_O_2_-stressed astrocytes	60.9, 182.7, 548.1 mg/kg/d in rats (*i.g.*), 0.3–3 μ g/mL for 96 h in cultured neurons, 1.5–15 μ g/mL for 48 h in cultured astrocytes	Promote neurogenesis and induced neurotrophic factors expression	Modification of Erk1/2 and CREB phosphorylation
Xian-He-Cao-Chang-Yan formula (Li et al., 2021a)	Agrimoniae Herba, Coptidis Rhizoma, Aucklandiae Radix, Cicadae Periostracum, ATR, and Platycodonis Radix	UC	Mice RAW 264.7 cells	DSS-induced UC LPS-stimulated RAW 264.7 cells	2.5, 5, 10 g/kg/d (*i.g.*) for mice 3, 0.8, 0.2 mg/mL for cells	Regulate macrophage polarization and inhibit glycolysis	The modulation of macrophage metabolic reprogramming *via* AMPK pathway
Longshengzhi capsule (Yin et al., 2020)	Hirudo, Astmgali Radix, Carthami Flos, Persicae Semen, ATR, and Acanthopanax Senticosus	AD	Mice HT-22 cells	Double transgenic mice expressing the APP/PS1 to model AD	850, 2000 mg/100 g food in mice 0, 10, and 20 μg/mL for 3 h in HT-22 cell	Anti-oxidative stress, anti-inflammatory and neuroprotective effects	In part by regulating the FAS/Bcl-2/p53 and NF-kB pathway
Smart Soup (Hou et al., 2014)	ATR, PR, PRP	AD	APPswe/PS1dE9 (APP/PS1) double-transgenic mice	Aβ-induced AD	10 g/kg (*i.g.*)	Improves cognitive impairment and reduces brain gliosis and neuronal loss in AD mice	Neuroprotective effects against Aβ

Notes: GR, ginseng radix; PR, ATR, acori tatarinowii rhizoma; PRP, poria cum radix pini; MDD, major depressive disorder; CUMS, chronic unpredictable mild stress; NGF, nerve growth factor; BDNF, brain-derived neurotrophic factor; GDNF, glial-derived neurotrophic factor; UC, ulcerative colitis; DSS, dextran sulfate sodium; LPS, lipopolysaccharide.

## Pharmacokinetics of ATR

Literature studies have found that there are relatively few pharmacokinetic studies on ATR and its active components. Ning et al. investigated the pharmacokinetic parameters of active components such as *cis*-methyl isoeugenol, β-asarone, α-asarone, and asarylaldehyde in ATR (2.16 g/kg, *p. o.*) in rats. The results indicated that β-asarone, α-asarone, *cis*-methylisoeugenol, and asarylaldehyde were absorbed slowly after oral administration of ATR (T_max_ = 4.78, 3.54, 3.51, and 2.21 h, respectively) (Ning et al., 2021) (Table 8). Due to the limited references reporting on the pharmacokinetic parameters of ATR, more studies need to be designed to further clarify the pharmacokinetic parameters of the absorption, distribution, metabolism and excretion processes of ATR as well as its active components.

**TABLE 8:** Pharmacokinetic parameters of ATR.

Compounds	Species	Dose of ATR	Pharmacokinetics parameter
*cis*-Methylisoeugenol (Ning et al., 2021)	Rats	2.16 g/kg ATR, *p.o*	C_max_ (ng/mL): 19.432 ± 18.798
			T_max_ (h): 3.514 ± 3.753
			T_1/2_ (h): 7.493 ± 2.55
			AUC_0-∞_ (ng·h/mL): 93.824 ± 20.379
			AUC_0-∞_ (ng·h/mL): 123.263 ± 30.025
β-Asarone (Ning et al., 2021)	Rats (Ning et al., 2021)	2.16 g/kg ATR, *p.o*	C_max_ (ng/mL): 49.752 ± 16.049
			T_max_ (h): 4.778 ± 3.777
			T_1/2_ (h): 10.662 ± 6.942
			AUC_0-∞_ (ng·h/mL): 603.44 ± 280.969
			AUC_0-∞_ (ng·h/mL): 1,045.247 ± 910.63
α-Asarone (Ning et al., 2021)	Rats (Ning et al., 2021)	2.16 g/kg ATR, *p.o*	C_max_ (ng/mL): 73.456 ± 25.933
			T_max_ (h): 3.542 ± 3.736
			T_1/2_ (h): 11.797 ± 12.574
			AUC_0-∞_ (ng·h/mL): 599.674 ± 360.384
			AUC_0-∞_ (ng·h/mL): 1,042.098 ± 614.716
Asarylaldehyde (Ning et al., 2021)	Rats (Ning et al., 2021)	2.16 g/kg ATR, *p.o*	C_max_ (ng/mL): 119.288 ± 52.23
			T_max_ (h): 2.208 ± 3.217
			T_1/2_ (h): 7.177 ± 1.232
			AUC_0-∞_ (ng·h/mL): 1,439.382 ± 492.857
			AUC_0-∞_ (ng·h/mL): 1,547.283 ± 482.098

Notes: ATR, acori tatarinowii rhizoma; C_max_, peak concentration of drug; T_max_, peak time; T_1/2_, half-life of elimination; AUC_0-∞_, area under the plasma concentration-time curve.

## Toxicity of ATR

Currently, the toxicological studies on ATR focus on its active components. The median lethal dose (LD_50_) of ATR volatile oil to mice was 0.22 ± 0.055 mL/kg. At the therapeutic dose, ATR volatile oil could significantly slow down the heart rate and prolong the P-R interval (Wu et al., 2009). Preliminary experiments found that the beating frequency of cardiomyocytes cultured *in vitro* was slowed down. Therefore, it is speculated that ATR volatile oil acts by inhibiting myocardial excitability and automaticity (Shen et al., 1993). It is worth noting that the existing research has proved that α-asarone and β-asarone may have carcinogenicity, teratogenicity and mutagenicity toxicity (Chellian et al., 2017). Therefore, it is necessary to pay attention to the contraindications and dosage of drugs. Simultaneously, the balance between curative effect and toxic and side effects should be grasped in clinical use. According to the literature reports, ATR has anti-cancer activity (Wu et al., 2004). Nevertheless, long term or high-dose toxicity testing in animals to explore the acute and chronic toxicity of ATR is still lacking. Thus, more meaningful studies need to be further designed to explore the toxicology of ATR.

## Conclusion and perspective

### Summary of evidence

As a traditional Chinese medicine in China, ATR has a long history of medicinal use and a wide range of pharmacological effects. ATR treatment of diseases has the characteristics of multi-component, multi-target, and multi-pathway synergy, among which there are many active monomer components, and the potential mechanism is complex and diverse. The volatile drugs with aromatic odor are administered through nasal inhalation and smell, which has become an effective way to prevent and treat diseases of the central nervous system (Zhang et al., 2021). The volatile oil of ATR is the main pharmacological component, and it has shown definite curative effect and good application prospect in the prevention and treatment of central nervous system diseases. Modern pharmacological studies have shown that ATR has an extremely wide range of pharmacological effects, and has a good preventive effect on a variety of diseases, especially central nervous system diseases, such as anti-depression, anti-AD, anti-Parkinson’s disease (PD), anti-epileptic, anti-cerebral ischemia-reperfusion injury and other effects, which has gradually become one of the hot studies in the field of medicine (Chellian et al., 2017).

At present, the central nervous system effect of ATR is mainly related to its regulation of cholinergic system, regulation of synaptic plasticity, antioxidation and protection of neurons. ATR is often used in combination with PR and GR in the treatment of central nervous system diseases. It is composed of KXS, PR powder and other well-known prescription for intelligence. It is the representative and basic prescription for future generations of prescription for intelligence. It has a good development and application prospect because of its high safety and obvious curative effect. However, since the pathogenesis of central nervous system diseases and the chemical components of ATR are relatively complex, more comprehensive and systematic research on the pathogenesis and pharmacological effects of central nervous system diseases should be strengthened in the future to provide more sufficient theoretical basis for ATR to treat central nervous system diseases.

α-Asarone and β-asarone are isomers of each other. Because of their volatile oil properties, α-asarone and β-asarone can quickly pass through the BBB to exert pharmacological effects on the central nervous system (Lam et al., 2019), and showing a very promising application prospect. Based on the aromatic odor of α-asarone and β-asarone and their easy penetration through the BBB, nasal inhalation and olfactory administration has opened up a new way for the volatile oil of ATR to prevent and treat central nervous system diseases, especially mental diseases. This is an efficient, low-toxic, safe and simple route of administration, which is worthy of in-depth research and exploration.

### Correlation between the chemical constituents and pharmacological mechanism of ATR

**FIGURE 3:**
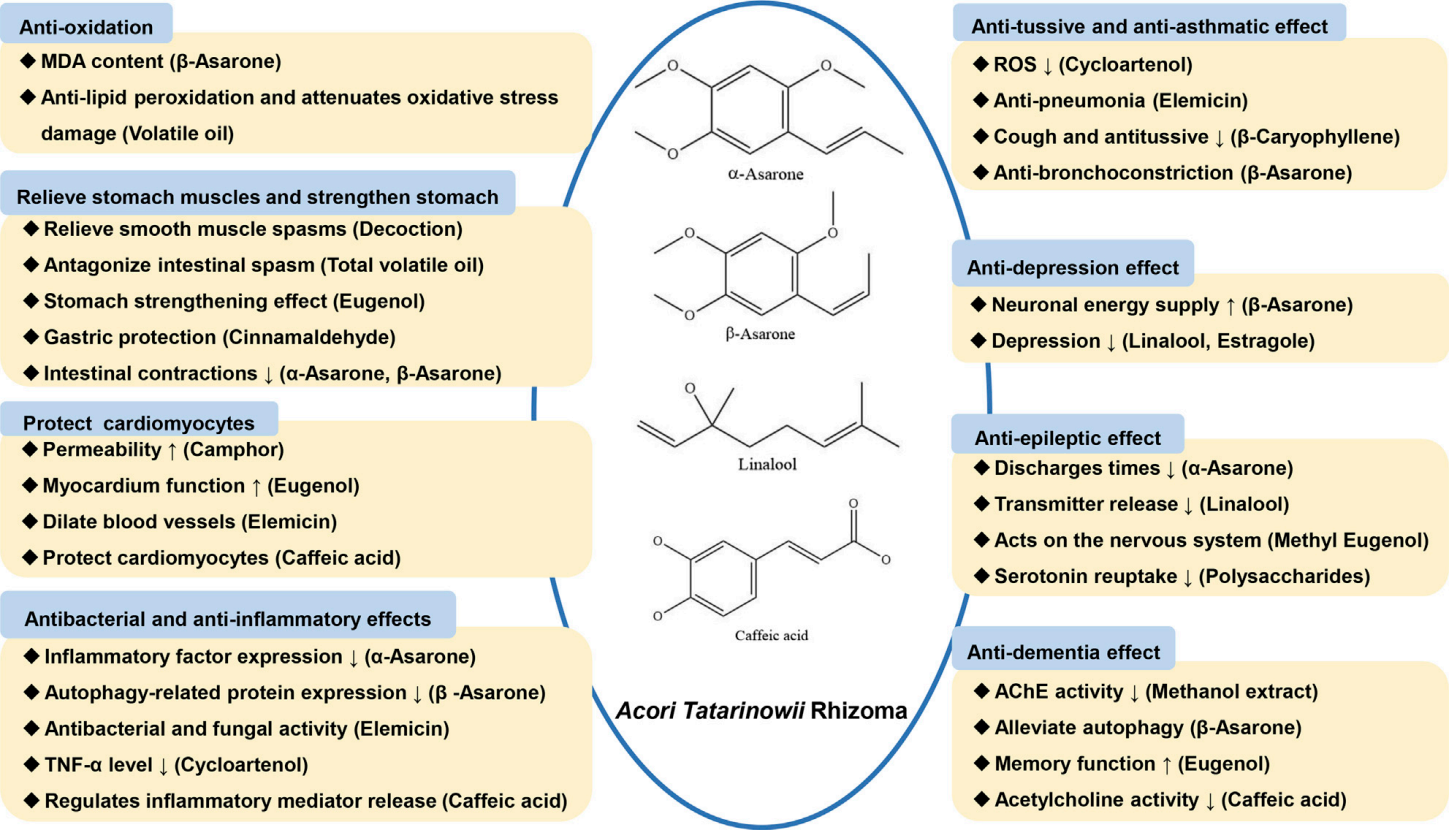
Active chemical constituents and pharmacological mechanism of ATR.

From this study, ATR can be used to prevent and treat central nervous system diseases, cardiovascular system diseases, gastrointestinal digestive system diseases, respiratory system diseases, etc. Therefore, this study summarizes the correlation between its chemical constituents and its pharmacological mechanism of action. A large number of experiments have shown that the volatile oil of ATR has a two-way regulation of excitation and inhibition on the central nervous system. It has antiarrhythmic, antithrombotic and protective effects on cardiac cells and blood vessels. Besides, ATR has anti-spasmodic and anti-asthmatic effects on the respiratory system and can promote digestion and regulate gastrointestinal movement for the digestive system. In terms of anti-epilepsy, α-asarone can reduce the number of discharges; linalool reduces transmitter release; methyl eugenol acts on the nervous system; polysaccharides inhibit serotonin reuptake. In terms of anti-depressant effects, β-asarone can improve behavioral disorders and increase neuronal energy supply. β-pinene can interact with monoamine systems. Linalool and artemisinin can reduce depression. Volatile oils and their aqueous extracts, methanol extracts, β-asarone, eugenol, caffeic acid, etc. can play an anti-dementia role by inhibiting β-amyloid aggregation and fiber formation, inhibiting acetylcholine esterase (AChE) activity, relieving autophagy, improving memory, and inhibiting acetylcholine activity, etc. In terms of cardiovascular activity, volatile oil, β-asarone, camphor, eugenol, elemene and caffeic acid have effect on reducing atherosclerotic blood lipids cholesterol (CHOL) and low-density lipoprotein cholesterol (LDL-C), enhancing permeability, exciting myocardium, dilating blood vessels, protecting cardiomyocytes. ATR water extract, total volatile oil, eugenol, cinnamic aldehyde, β-asarone, α-asarone block the receptors, relieve smooth muscle spasm, antagonize intestinal spasm, protect gastric function, inhibit intestinal contraction, etc. Gastrointestinal muscles and play a role in stomach. For the respiratory system, ATR can inhibit the generation of ROS, increase tracheal secretion, dilute sputum, inhibit bronchoconstriction, relax tracheal smooth muscle, block MAPK and NF-κB to exert anti-tussive and asthmatic effects. Other chemical basis and pharmacological mechanism of action are shown in Figure 3.

### Research limitations and problems

Currently, the research on ATR mainly focuses on the volatile components. The traditional usage is to boil it into a water decoction, and its volatile components will be lost (Yang et al., 2021). Whether and to what extent the loss of volatile components of ATR and related preparations has an impact on the quality of the drug is unknown. Some studies have reported that its volatile components are present in both oil and water media. Whether these two media are the same and what are the similarities and differences when they exert their medicinal effects is worthy of further study. There are various studies on the volatile components of ATR, such as α-asarone and β-asarone, but less research on its non-volatile components. Thus, there are still insufficient studies on the systemic activity and pharmacological mechanism, active ingredients, pharmacokinetic characteristics of volatile and non-volatile components of ATR. Besides, the qualitative and quantitative analysis of various chemical components in ATR still needs to be detected and analyzed by ultrahigh performance liquid chromatography quadrupole time of flight mass spectrometry (UHPLC/Q-TOF-MS). In addition, there are few reports on pharmacokinetics and toxicology of ATR and its active components in the current study. Therefore, a more comprehensive and in-depth excavation of the effect of ATR will promote its new clinical use, thereby providing useful guidance for improving its medicinal value. The further study can combine ATR with modern pharmaceutical technology to explore whether it can be used as a drug carrier to assist chemical medicine to achieve the leap of BBB, reduce the dosage of chemical medicine, reduce adverse reactions, and make ATR more widely applicable. It is expected to provide scientific basis for the promotion and application of ATR in the prevention and treatment of various diseases, and can become an antiepileptic drug with significant clinical efficacy, low toxicity and more safety.

As a common traditional Chinese medicine, ATR has a long history of application in China, and is widely used in the treatment of diseases of the central nervous system, cardiovascular system, digestive system and respiratory system. However, the chemical components, pharmacological activity, toxicological effect and molecular mechanism of ATR need to be further explored. The future study should focus on clarifying the chronic toxicity and acute toxicity of ATR to further clarify its safety, so as to guide the clinical rational use of drugs and the development of new drugs. In addition, nanotechnology and chemical modification can improve the oral bioavailability of ATR and expand the scope of drug clinical treatment.

## Glossary

ATR: Acori Tatarinowii Rhizoma
CNKI: China National Knowledge Infrastructure
VMIS: VIP medicine information system
CBM: Chinese Biomedical Database
ATEO: The essential oil of ATR
NMR: Nuclear magnetic resonance
AD: Anti-Alzheimer’s disease
BBB: Blood-brain barrier
NGF: Nerve growth factor
BDNF: Brain-derived neurotrophic factor
GDNF: Glial-derived neurotrophic factor
Aβ: Amyloid beta
tBHP: Tert-butyl hydroperoxide
ROS: Reactive oxygen species
ARE: Anti-oxidant response element
CREB: cAMP-response element binding protein
SOD: Superoxide dismutase
NO: Nitric oxide
NPC: Neural progenitor cell
ERK: Extracellular signal-regulated kinase
APP: Amyloid precursor protein
ESR1: Estrogen receptor 1
PPARG: Peroxisome proliferator activated receptor gamma
AR: Androgen receptor
CHRM1: Muscarinic acetylcholine receptor M1
CASP3: Caspase 3
JAK2: Janus kinase 2
MAPK14: Mitogen-activated protein kinase 14
PTGS1: Prostaglandin G/H synthase 1
PTPN1: Protein tyrosine phosphatase non-receptor type 1
KDR: Kinase domain receptor
ET: Endothelin
CGRP: Calcitonin gene-related peptide
NE: Norepinephrine
MDA: Malondialdehyde
CK: Creatine kinase
MI/RI: Myocardial ischemia/reperfusion injury
LDH: Lactate dehydrogenase
MMP: Mitochondrial membrane potential
EMT: Epithelial-mesenchymal transition
Bax: Bcl2-associated X
Bak: Bcl-2 homologous antagonist/killer
PDK: Pyruvate dehydrogenase kinase
Ach: Acetylcholine
Hist: Histamine phosphate
TMCA: 3,4,5-trimethoxycinnamic acid
PR: Polygalae Radix
P-gp: P-glycoprotein
TNF-α: Tumor necrosis factor alpha
NF-kB: Nuclear factor kappa B
LPS: Lipopolysaccharide
KXS: Kaixin San
XHCYF: Xian-He-Cao-Chang-Yan formula5
LSZ: Longshengzhi capsule
GR: Ginseng Radix
PRP: Poria cum Radix Pini
TLR4/IKK/NF-κB: Toll-like receptor 4/inhibitor of kappa B kinase/nuclear factor kappa-B
CMS: Chronic mild stress
CUMS: Chronic unpredictable mild stress
NGF: Nerve growth factor
Trk: Tropomyosin receptor kinase
XHCF: Xian-He-Cao-Chang-Yan formula
DSS: Dextran sulfate sodium
UC: Ulcerative colitis
AMP: Adenosine 5‘-monophosphate
AMPK: Activated protein kinase
APP: Amyloid-β precursor protein
PS1: Presenilin 1
PD: Anti-Parkinson’s disease
AChE: Acetylcholine esterase
CHOL: Cholesterol
LDL-C: Low-density lipoprotein cholesterol.
